# Bee Venom: Overview of Main Compounds and Bioactivities for Therapeutic Interests

**DOI:** 10.3390/molecules24162997

**Published:** 2019-08-19

**Authors:** Rim Wehbe, Jacinthe Frangieh, Mohamad Rima, Dany El Obeid, Jean-Marc Sabatier, Ziad Fajloun

**Affiliations:** 1 Laboratory of Applied Biotechnology (LBA3B), Azm Center for Research in Biotechnology and its Applications, EDST, Lebanese University, Tripoli 1300, Lebanon; 2 Mitochondrial and Cardiovascular Pathophysiology—MITOVASC, Team 2, Cardiovascular Mechanotransduction, UMR CNRS 6015, INSERM U1083, Angers University, 49045 Angers, France; 3 Department of Neuroscience, Institut de Biologie Paris Seine (IBPS), INSERM, CNRS, Sorbonne Université, F-75005 Paris, France; 4 Institut de Génétique et de Biologie Moléculaire et Cellulaire (IGBMC), INSERM U964, CNRS U7104, Université de Strasbourg, 67400 Illkirch, France; 5 Faculty of Agriculture & Veterinary Sciences, Lebanese University, Dekwaneh, Beirut 2832, Lebanon; 6 Institute of NeuroPhysiopathology, UMR 7051, Faculté de Médecine Secteur Nord, 51, Boulevard Pierre Dramard-CS80011, 13344-Marseille Cedex 15, France; 7 Faculty of Sciences 3, Michel Slayman Tripoli Campus, Lebanese University, Ras Maska 1352, Lebanon

**Keywords:** bee venom, apitoxin, apitherapy, Parkinson’s disease, Alzheimer’s disease, amyotrophic lateral sclerosis, cancer, HIV

## Abstract

Apitherapy is an alternate therapy that relies on the usage of honeybee products, most importantly bee venom for the treatment of many human diseases. The venom can be introduced into the human body by manual injection or by direct bee stings. Bee venom contains several active molecules such as peptides and enzymes that have advantageous potential in treating inflammation and central nervous system diseases, such as Parkinson’s disease, Alzheimer’s disease, and amyotrophic lateral sclerosis. Moreover, bee venom has shown promising benefits against different types of cancer as well as anti-viral activity, even against the challenging human immunodeficiency virus (HIV). Many studies described biological activities of bee venom components and launched preclinical trials to improve the potential use of apitoxin and its constituents as the next generation of drugs. The aim of this review is to summarize the main compounds of bee venom, their primary biological properties, mechanisms of action, and their therapeutic values in alternative therapy strategies.

## 1. Generalities about Honeybees

**Figure 1:**
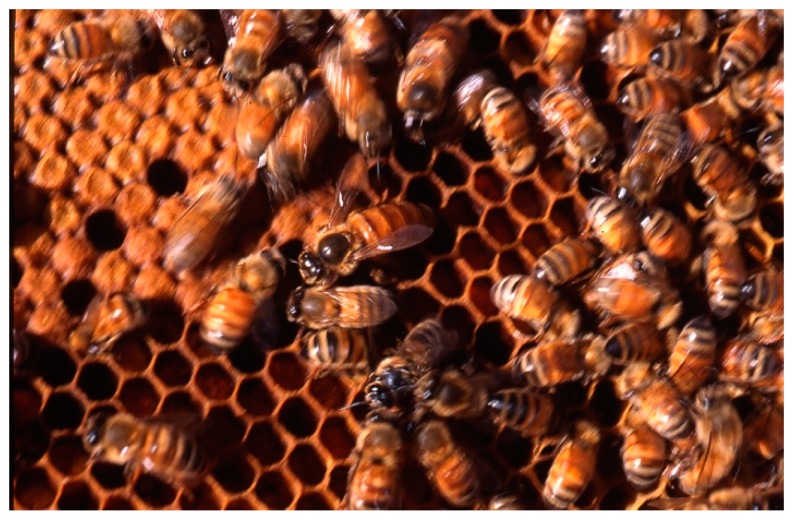
*Apis mellifera* (copyright Dany El Obeid).

Among honeybees, *Apis mellifera* (Figure 1) is the main species used for crop pollination in the world [1]. The usage of all bee products, including bee venom and honey, dates back thousands of years as their medicinal properties were cited in religious books like the Bible and the Quran [2,3,4]. Apitherapy is a branch of alternative medicine that relies on the usage of honeybee products that consists of honey, pollen, propolis, royal jelly, and mainly bee venom (BV), which is also known as apitoxin [5,6].

**Figure 2:**
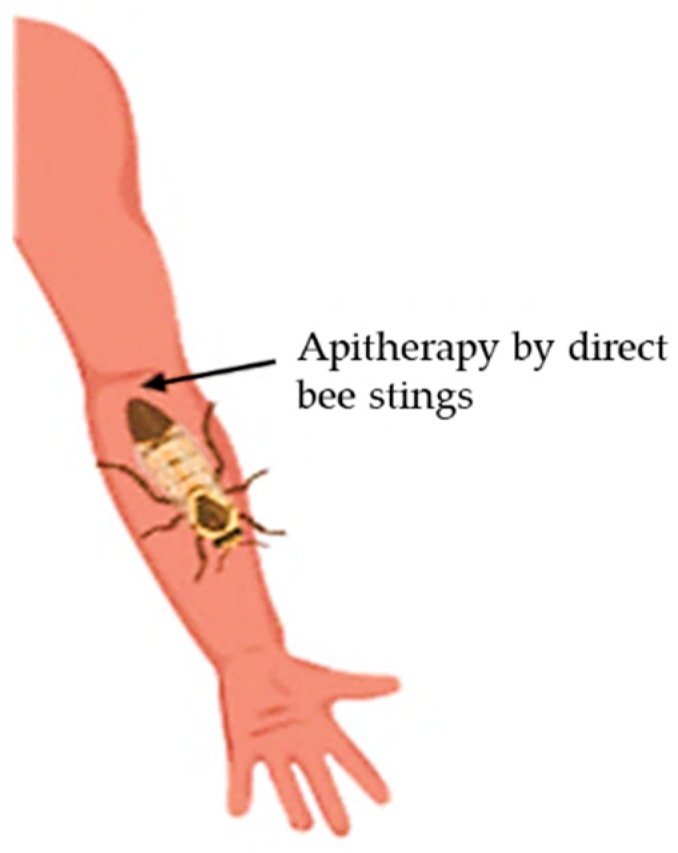
Application of bee venom by direct bee stings into the body.

Bee venom therapy (BVT) is the medicinal application of BV from honeybees into the human body for the treatment of some diseases, such as rheumatism arthritis [7]. This strategy has been used in alternative medicine for more than 5000 years. It consists of either indirect application, by extracting BV with an electric stimulus followed by its injection into the body or directly via bee stings [8] (Figure 2). The idea of using BV in the medicinal field was raised from the belief that beekeepers hardly suffer from rheumatism or joints problems.

BV is produced by female worker bees and is known to contain many active components including: (i) peptides like melittin, apamin, mast cell degranulating (MCD) peptide, and adolapin, (ii) enzymes, such as phospholipase A2 (PLA2) and hyaluronidase, and (iii) amino acids and volatile compounds. Several studies assessed the therapeutic potential of these components in treating human inflammatory diseases as well as central nervous system diseases, such as Parkinson’s disease (PD), Alzheimer’s disease (AD), and amyotrophic lateral sclerosis (ALS), as well as many other conditions [9,10]. Interestingly, bee venom, in similarity to other animal venoms, has also shown beneficial anti-cancer and anti-viral potential against ovarian and prostate cancer, as well as HIV [11,12,13,14].

Bee venom is characterized by inducing allergic reactions following the sting. These reactions can take place in the skin, the respiratory track, the cardiovascular system, and the gastrointestinal system. Subsequently, severe anaphylactic shock could lead to cerebral or myocardial ischemia [15,16]. These allergic responses are due to the presence, within the venom, of multiple protein allergens, most of which possess an enzymatic activity [9]. The major BV allergens and specific Immunoglobulin E (IgE) inducers are PLA2, melittin, and hyaluronidase. Apart from IgE-mediated mechanisms, studies suggest that allergens can also involve IgE-independent reactions, such as a bradykinin (BK) mediator, leading to various anaphylactic symptoms [17,18]. The production of this non-immune mediator can be induced by melittin, known as a PLA2 activator that can mimic BK’s effects on tracheal tone [17,19]. In addition, MCD-peptide or peptide 401 is able to induce an anaphylactoid reaction by degranulating mast cells [9,20].

Beside molecular studies investigating the possible mechanisms behind inflammatory bee sting responses, many clinical studies are deeply looking into the potential use of BV for treating chronic diseases. Hence, the following parts of the review aim to highlight the primary biological properties of BV and its bioactive molecules that have potential in developing therapeutic strategies.

## 2. Main Compounds of Bee Venom

BV is an odorless and transparent liquid containing a hydrolytic mixture of proteins with acid pH (4.5 to 5.5) that bees often use as a defense tool against predators. One drop of BV consists of 88% of water and only 0.1 µg of dry venom [10]. The latter is an extremely complex blend of peptides including melittin, adolapin, apamin, and MCD-peptide. It also contains enzymes, most importantly PLA2, and compounds of low molecular weight like bioactive amines (e.g., histamine and epinephrine) and minerals [9].

### 2.1. Melittin

Melittin, a 26-residue peptide, is the main component of BV and accounts for 40–60% of its composition [21]. The carboxyl-terminal region of the peptide is hydrophilic and responsible for the lytic action, while the amino-terminal region of its sequence is predominantly hydrophobic with no lytic activity [22]. The amphipathic property of melittin makes it soluble in water in both its monomeric and tetrameric forms. It also allows melittin to be easily inserted into membranes by disrupting both natural and synthetic phospholipid bilayers. Previous studies have shown that the mechanism of action of melittin in disrupting membranes is mediated by pore formation lysing both prokaryotic and eukaryotic cells in a non-selective matter. In fact, melittin binds to membranes as monomers but acts on the membrane inclusively. Depending on its concentration, this biopeptide can induce either transient or stable pores [23]. When a transient pore is formed, only ions are able to diffuse through the membrane. However, in the case of stable pore formation, the membrane becomes permeable to relatively large molecules, such as glucose [24]. The pore formation induced by melittin is responsible of its hemolytic, anti-microbial, anti-fungal, and antitumor activities [12,25]. Lately, melittin has been shown to cause neural plastic changes along pain-signaling pathways by activation and sensitization of nociceptor cells. The mechanism involves the phosphorylation of mitogen-activated protein kinases (MAPK) as well as the activation of thermal nociceptive channels like TRPV1 (transient receptor potential vanilloid receptor 1), ATP-gated P2X and P2Y purinergic receptors. Likewise, melittin can act as an activator of PLA2 [26]. It is also a major biologically active substance of BV that produces anti-nociceptive, anti-inflammatory, and anti-arthritic effects once administrated to the acupoint of the patient [27].

### 2.2. Apamin

Apamin is an 18-amino acid peptide containing two disulfide bridges. It is the smallest neurotoxin in BV [28]. This polypeptide is able to cross the blood-brain barrier and therefore it affects the central nervous system functioning via different modes of action. For example, it causes neurotoxic effects in the mammalian spinal cord, resulting in hyperactivity and seizures, as it has been shown in rats. By blocking calcium-activated K^+^ channels, apamin is also able to affect the permeability of cell membrane toward potassium ions (K^+^). In the vascular smooth muscle, the toxin is able to inhibit vascular smooth muscle cell proliferation and migration via the Akt and Erk signaling pathways [29]. This finding highlights the potential of apamin in atherosclerosis therapy strategies. Another study assessed the consequences of K^+^ channels sensitivity to apamin and showed that the neurotoxin can inhibit NO-induced relaxation of the spontaneous contractile activity of the myometrium in non-pregnant women [30].

### 2.3. Mast Cell Degranulating (MCD) Peptide

The MCD peptide, also known as peptide 401, is a BV polypeptide containing 22 amino acids with similar structure to apamin, as they both contain two disulfide bonds. It accounts for 2–3% of BV’s dry weight. The name MCD echoes the biological action in histamine release from mast cells. It is an epileptogenic neurotoxin, an important inhibitor of K^+^ channels, and can cause a significant reduction in the blood pressure of rats [31]. Some of MCD biological activities seem to have distinct mechanisms and may represent a good illustration of the structure-function relationship. Studies describe MCD as a powerful anti-inflammatory agent and may serve as a potential candidate for the study of secretory mechanisms of inflammatory cells, such as mast cells, basophils, and leukocytes, leading to the design of compounds with therapeutic applications [32].

### 2.4. Adolapin

Adolapin is a basic polypeptide with 103 amino acids residues. It corresponds to 1% of the dry weight of BV. Researchers have shown that adolapin possesses anti-inflammatory, anti-nociceptive, and antipyretic effects by blocking prostaglandin synthesis and inhibiting cyclooxygenase activity [33]. The polypeptide can inhibit lipoxygenase from human platelets and may exert an analgesic effect according to Jung et al. [34].

### 2.5. Phospholipase A_2_

PLA2, the most lethal enzyme in BV, is a single polypeptide chain of 128 amino acids containing four disulfide bridges. Bee venom phospholipase A2 (bvPLA2) belongs to the group III sPLA_2_ enzymes and can act as a ligand for specific receptors. BvPLA2 represents 12–15% of BV’s dry weight and is extremely alkaline. BvPLA2 is a hydrolytic enzyme, able to specifically cleave the sn-2 acyl bond of phospholipids at the water/lipid interface [35]. Interestingly, its activity can be improved by melittin. This has been shown to occur during the process of erythrocyte lysis, proving the presence of synergistic action between both bvPLA_2_ and melittin [36,37]. In fact, it has been demonstrated that melittin helps in exposing membrane phospholipids to the catalytic site of enzymes via opening melittin-induced channels [38]. Additionally, new experimental data have demonstrated protective immune responses of bvPLA_2_ against a broad range of diseases, such as asthma, Alzheimer’s disease, and Parkinson’s disease [39,40,41]. BvPLA2 plays a neuroprotective role by inducing the microglial deactivation and reducing CD4^+^ T cell infiltration in the MPTP-induced mouse model of PD (MPTP: 1-methyl-4-phenyl-1,2,3,6-tetrahydropyridin) [42].

### 2.6. Hyaluronidase

Hyaluronidase represents 1.5–2% of BV dry weight and is known to break down hyaluronic acid in tissues, such as in synovial bursa in rheumatoid arthritis. BV hyaluronidase allows the active components of BV to diffuse effectively into a victim’s tissue by affecting its structural integrity and increasing blood flow in the area. These two actions combine to intensify the wide spreading of the venom [43,44].

## 3. Bioactivities and Therapeutic Applications of Bee Venom and Its Major Compounds

### 3.1. Anti-Inflammatory Potential

Inflammation is a protective process for the body in response to harmful stimuli. Chronic inflammation can lead to the development of several diseases like rheumatoid arthritis (RA), diabetes, cardiovascular disease, obesity, asthma, skin diseases, and CNS-related diseases, such as PD, AD, and ALS [45].

Melittin, when administrated at high doses, causes local pain, itching, and inflammation. However, low doses of this BV compound can induce wide anti-inflammatory effects. Many reports investigated the anti-inflammatory mechanisms of melittin in different diseases such as RA and ALS [46,47]. In fact, it acts by inhibiting inflammatory cytokines like interleukin-6 (IL-6), IL-8, tumor necrosis factor-α (TNF-α), and interferon-γ (IFN-γ). Moreover, melittin decreases signaling pathways that activate inflammatory cytokines, including nuclear factor-kappa B (NF-κB), protein kinase Akt, and extracellular signal-regulated kinases (ERK1/2) in porphyromonas gingivalis lipopolysaccharide (PgLPS)-treated human keratinocytes. These findings indicate that, by blocking their primary signaling pathways, melittin inhibits inflammatory cytokines leading to a reduced inflammation in skin, liver, joint, and neuronal tissue [48].

Regarding skin diseases, a recent study by Kim et al. showed that BV reduces Atopic Dermatitis, the most common allergic chronic inflammatory skin disease [49]. In fact, the venom stimulates CD55 production by triggering ERK1/2 pathways, which leads to the alleviation of the disease’s symptoms [50]. Interestingly, a previous study by Shin et al. described the anti-inflammatory potential of bvPLA2 in skin diseases by showing that the enzyme attenuates Atopic skin inflammation through interaction with CD206 [51].

### 3.2. BV Application for the Treatment of Neurodegenerative Diseases

#### 3.2.1. Parkinson’s Disease

PD is a degenerative movement disorder that leads to progressive disability in patients. The pathological hallmarks of the disease are the progressive loss of dopaminergic neurons in the substantia nigra (a basal ganglia structure found in the human brain), and the presence of Lewy bodies that contains aggregates of alpha-synuclein, a widely distributed protein in the brain [52,53]. Abnormal microglial activation is also a pathological sign in different neurodegenerative diseases, including PD [54]. Many preclinical trials investigated the effect of BV on the migration of leukocytes or microglial activation in animal and cellular models. Other tests evaluated the neuroprotective potential of BV acupuncture therapy (BVA) against rotenone-induced oxidative stress, neuroinflammation, and apoptosis in PD mice models [55]. Rotenone is a pesticide that may affect pathophysiological mechanisms that are implicated in PD [55]. Interestingly, BV proved its ability to prevent dopamine depletion after the administration of rotenone. In addition, the locomotor activity was re-established after treating PD mice models with BV. The treatment effectively repressed DNA damage and inhibited the expression of the apoptotic Bax, Bcl-2, and caspase-3 genes in the brain of PD mice. These findings demonstrate that BV normalized all the apoptotic and neuroinflammatory markers and restored brain neurochemistry after rotenone injury [56]. It has also been shown that BV can protect doparminergic neurons from degeneration in experimental PD models. Along with this finding, acupoint stimulation of lower hind limbs with BV was found to be protective in the MPTP (1-Methyl-4-Phenyl-1,2,3,6-TetrahydroPyridine) mouse model of PD [57].

#### 3.2.2. Alzheimer’s Disease

AD is the most common neurodegenerative disease and many pathological processes are involved in its emergence [58]. However, the hypothesis of amyloid cascade and the toxicity of amyloid beta (Aβ) peptides have dominated research so far due to advanced studies showing that aggregates of this peptide are characteristic signs of the disease [59,60,61]. Although the etiology of AD remains unknown, the evidences suggest that inflammatory responses may play a crucial role in its pathogenesis [62,63]. The current treatments for cognitive loss related to AD rely on the usage of muscarinic or nicotinic receptor ligands and the acetylcholinesterase (AChE) inhibitor [64]. As an alternative strategy, Ye et al. showed that bvPLA2 can be used as a treatment to block the progression of AD in transgenic mice [40]. This is due to the ability of bvPLA2 to reduce the accumulation of Aβ and improve cognitive functioning in mice brains. The same study similarly shows that bvPLA2 can increase glucose brain metabolism and reduce neuroinflammatory responses in the hippocampus, which can limit AD pathogenesis [40]. A recent study also showed that regulatory T-cells populations could be modulated by bvPLA2 treatment in a 3xTg-AD mouse model. Therefore, authors suggested a new therapeutic approach to reduce the progression of AD by combining bvPLA2 treatment along with Aβ vaccination therapy to prevent its adverse inflammatory response [60].

#### 3.2.3. Amyotrophic Lateral Sclerosis

ALS is a CNS disease that causes the death of motor neurons [65]. A significant trait of ALS is the abnormal accumulation of mutant SOD1 (mtSOD1) protein aggregates [66]. A mice model of ALS carrying the mutated mtSOD1 gene with a Glycine to Alanine substitution (SOD1^G93A^) was characterized by Jaarsma et al., facilitating the understanding of ALS etiology [67]. Both in vitro and in vivo studies using the mutant SOD1 transgenic mice demonstrated various cellular pathogenic events in motor neurons like protein misfolding, dysfunction of mitochondria, and accumulation of neurofilament [67]. Interestingly, BV showed some potential for counteracting this disease. In fact, the administration of BV, at a precise and symptomatic stage of the progression of ALS, leads to an increase in motor activity in SOD1^G93A^ mutant mice and a prolongation in life expectancy when compared with age-matched control mice. This could be caused by the blockage of activated microglia usually found in mice models of ALS [68]. Another study demonstrated that bee venom acupuncture (BVA) at ST36 inhibits neuroinflammation in the spinal cord of symptomatic ALS mice by significantly reducing the levels of inflammatory proteins like TLR4, CD14, and TNF-α [69].

### 3.3. BV and/or Melittin Applications in Cancer

**Figure 3:**
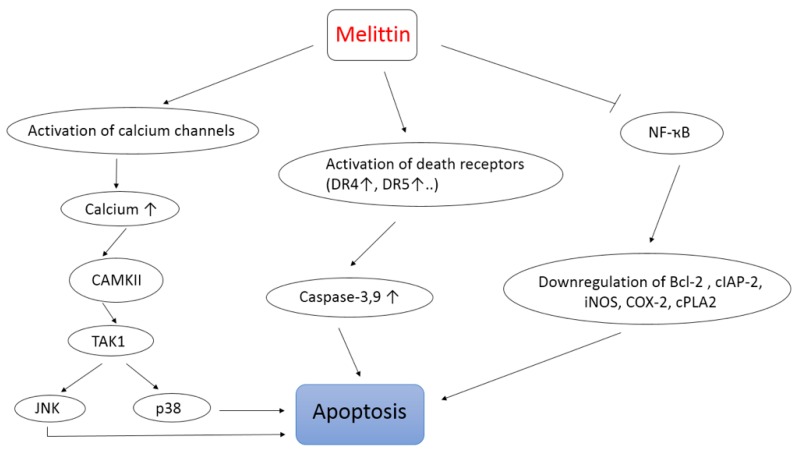
Schematic drawing of main mechanisms of action of melittin as an anti-cancer agent.

The use of apitoxin, especially its main compound melittin, as a novel cancer-treatment strategy has gained wide importance recently [70,71]. In fact, melittin is known to be a nonspecific cytolytic peptide that can attack the lipid bilayer, thus leading to a significant toxicity when injected intravenously [72]. Nevertheless, many optimization approaches, including the use of nanoparticle-based delivery of melittin, have been exploited. Remarkably, the crude BV as well as melittin have shown antitumor activities against different cancer cell types including breast, liver, leukemia, lung, melanoma, and prostate cancer cells [70,72,73,74,75]. Wang et al. [76] investigated the mechanism behind melittin antitumor activity and showed that melittin can induce apoptosis of hepatocellular carcinoma cells (HCC) through the activation of the CAMKII-TAK1-JNK/p38 signaling pathway (CAMKII: Ca^2+^/calmodulin-dependent protein kinase; TAK1: Transforming growth factor-beta-activated kinase 1; JNK/p38: Mitogen-activated protein kinases). Moreover, melittin can sensitize TRAIL-resistant HCC cells (TRAIL: Tumor necrosis factor-related apoptosis-inducing ligand) to TRAIL-induced apoptosis, probably via activating the CAMKII-TAK1-JNK/p38 pathway and inhibiting the IKK-NFκB pathway (Figure 3). These findings are in agreement with the activation of calcium channels by melittin that leads on to the increase of intracellular Ca^2+^ concentration and the activation of calcium sensitive CaMKII, as seen also in Figure 3 [76].

Park et al. [13] also reported that BV and its major component, melittin, induce an inhibition of cancer cells growth both in vitro and in vivo via the activation of caspases (3 and 9) pathways and the inhibition of NF-κB signaling and its downstream proliferative and anti-apoptotic gene products like Bcl-2, cIAP-2, iNOS, COX-2, and cPLA2 (Figure 3) [13]. Similarly, Zheng et al. [77] demonstrated that BV exerts an anti-proliferative effect and induces apoptosis via the activation of death receptors (DR4 and DR5). Another interesting finding emerged about melittin by highlighting its anti-metastatic and antigrowth properties [73]. In cancer, metastasis and the invasion of malignant cells are the main reasons behind the progression of the disease. Therefore, researchers in the cancer field focus on understanding the molecular mechanisms that regulate malignant cell migration and the possible way to prevent it, as a crucial step in their fight against cancer [78,79]. In this context, it has been found that melittin inhibits in vitro and in vivo HCC cells motility by suppressing Rac1-dependent pathways [73]. On the other hand, a recent study proved that the combination of melittin with a chemotherapeutic agent like temozolomide remarkably decreases growth along with the invasion of melanoma cells, compared to conditions where TMZ or melittin were used alone [71].

These findings show the great potential of melittin in cancer treatment by acting on different key points of the disease and should be further dissected.

Despite the convincing data regarding the potential use of BV, more specifically melittin, against a variety of cancer types, its applicability to humans remains very challenging because of its non-specific cytotoxicity [80]. Current optimization methods are focusing on nanoparticle-based delivery of melittin in order to avoid such problems. Due to nanotechnology, it has been possible to develop and effectively test conjugates of melittin against a broad range of human cancer types in preclinical models [81]. Cheng et al. aimed to develop an efficient yet safe delivery system for melittin, which can reduce its hemolytic activity while conserving its cytotoxic advantages. Therefore, a dual secured nano-sting (DSNS) was designed via the combination of a zwitterionic glycol chitosan and disulfide bonds. Melittin loaded DSNS showed almost complete cytotoxic effect on many cancer cells types at very low concentrations while leaving red blood cells unharmed [82]. Furthermore, it has been shown that intravenous administration of melittin prodrug-loaded nanoparticles, using per fluorocarbon nanoparticles, in a melanoma mouse model efficiently reduced the tumor growth rate compared to saline and blank nanoparticle treatment [83].

### 3.4. Antiviral and Antibacterial Properties

It is well known that BV and its two major components (melittin and PLA2) present antimicrobial activities and thus can be used as complementary anti-bacterial agents [84,85,86,87]. These compounds exert their effects against bacteria by inducing pores through their membranes leading to their cleavage and then lysis [36].

Nevertheless, the antiviral effect of BV has not been mentioned much in literature. A recent study investigated BV antiviral potential and came out with interesting findings both in vivo and in vitro. This study showed that BV and melittin have significant antiviral effects against numerous enveloped viruses (vesicular stomatitis virus, influenza A virus, herpes simplex virus, etc.) and non-enveloped viruses (enterovirus-71 and coxsackie virus) in vitro [88]. The study also showed that melittin protected mice that were exposed to lethal doses of influenza A H1N1 virus. Although the precise mechanism of action by which BV and melittin act as antiviral agents remains unclear, it has been confirmed that BV interacts directly with the viral surface. Moreover, BV and its components can stimulate type I interferon (IFN), and therefore suppress viral replication in the host cell [89].

Additionally, researchers at Washington University School of Medicine in St. Louis have reported the possible application of nanoparticles loaded with melittin in destroying the human immunodeficiency virus while leaving non-infected cells unharmed. In this approach, the authors suggest a preventive strategy in which these nanoparticles are used in developing a vaginal gel that inhibits the spread of HIV. Its theoretical principle is as follows: Melittin molecules present on nanoparticles fuse with the viral envelope forming pore-like attack complexes, thus breaking the viral envelope [14]. Another study showed that bvPLA2 can also block the replication of the virus. The same team further identified the peptide sequence of bvPLA2 responsible of the inhibition of HIV replication [89,90,91,92].

## 4. Conclusions

The use of BV for medical applications can be traced back thousands of years. Here, the therapeutic interests of crude bee venom and/or its main compounds, particularly melittin, are discussed. The latter grants broad anti-inflammatory properties by affecting primary inflammation signaling pathways and inducing the inhibition of pro-inflammatory genes expression. BV also possesses a neuroprotective potential in neurodegenerative diseases such as PD, AD, and ALS by significantly blocking their progression and improving cognitive functioning in mice models. In terms of antitumor activity, both melittin and BV have a cytotoxic effect on cancer cells and a significant anti-metastatic activity. Optimization approaches are currently focusing on the possible use of nanoparticle-based delivery of melittin, or even BV, in order to avoid their nonspecific cytotoxic effect. The antiviral activity of BV is also promising since BV and melittin have notable toxic effects against a broad spectrum of enveloped viruses, including the challenging HIV, and few non-enveloped viruses. Finally, the clinical application of BV therapy is still a long way ahead, but researchers believe that the ongoing work on this topic will eventually allow BV and its compounds to be considered as definitive candidates in various therapies in upcoming years.
